# Impact of Gene Polymorphisms in GAS5 on Urothelial Cell Carcinoma Development and Clinical Characteristics

**DOI:** 10.3390/diagnostics10050260

**Published:** 2020-04-28

**Authors:** Wei-Chun Weng, Chih-Jung Chen, Pei-Ni Chen, Shian-Shiang Wang, Ming-Ju Hsieh, Shun-Fa Yang

**Affiliations:** 1 Division of Urology, Department of Surgery, Tungs’ Taichung MetroHarbor Hospital, Taichung 435, Taiwan; 2 Department of Nursing, Jen-Teh Junior College of Medicine, Nursing and Management, Miaoli 356, Taiwan; 3 Department of Pathology and Laboratory Medicine, Taichung Veterans General Hospital, Taichung 407, Taiwan; 4 School of Medicine, Chung Shan Medical University, Taichung 402, Taiwan; 5 Institute of Biochemistry, Microbiology and Immunology, Chung Shan Medical University, Taichung 402, Taiwan; 6 Division of Urology, Department of Surgery, Taichung Veterans General Hospital, Taichung 407, Taiwan; 7 Department of Applied Chemistry, National Chi Nan University, Nantou 545, Taiwan; 8 Institute of Medicine, Chung Shan Medical University, Taichung 402, Taiwan; 9 Cancer Research Center, Changhua Christian Hospital, Changhua 500, Taiwan; 10 Graduate Institute of Biomedical Sciences, China Medical University, Taichung 404, Taiwan; 11 Department of Medical Research, Chung Shan Medical University Hospital, Taichung 402, Taiwan

**Keywords:** urothelial cell carcinoma, growth arrest-specific 5, polymorphism, susceptibility

## Abstract

Urothelial cell carcinoma (UCC) is the commonest malignant tumor of the urinary tract and the second most common kidney cancer malignancy. Growth arrest-specific 5 (GAS5), a long noncoding RNA, is encoded by the GAS5 gene and plays a critical role in cellular growth arrest and apoptosis. In the current study, two single nucleotide polymorphisms (SNPs) in the GAS5 gene, rs145204276 and rs55829688, were selected to investigate correlations between these single SNPs and susceptibility to UCC. A total of 430 UCC cases and 860 ethnically matched healthy controls were included. SNP rs145204276 and SNP rs55829688 were determined using a TaqMan genotyping assay. Logistic regression models demonstrated that female patients with UCC carrying the rs145204276 GAS5 Ins/Del or Del/Del genotype had a 3.037-fold higher risk of larger tumor status (95% confidence interval 1.259–7.324) than did rs145204276 wild type (Ins/Ins) carriers (*p*  =  0.011). The Cancer Genome Atlas validation cohort analysis demonstrated that the expression of GAS5 in female patients with bladder urothelial carcinoma (BLCA) with larger tumor size was much lower than that in patients with a smaller tumor size (*p* = 0.041). Kaplan-Meier curve analysis and the log–rank test revealed that female patients with BLCA and lower GAS5 expression had poorer overall survival than those with higher GAS5 expression. In conclusion, genetic variations in GAS5 rs145204276 may serve as a critical predictor of the clinical status of female patients with UCC.

## 1. Introduction

Urothelial cell carcinoma (UCC), also called transitional cell carcinoma, typically involves the urinary system and can develop in the lining of epithelial cells of the urethra, ureters, renal pelvis, or bladder [1]. UCC is the predominant histologic type in the United States, accounting for more than 90% of all bladder cancers; approximately 25% of patients with bladder cancer have muscle-invasive disease [2]. UCC of the bladder represents the fifth most commonly diagnosed malignancy in the United States [3], and patients with bladder cancer usually present with painless hematuria. Clinical delays in the diagnosis of bladder cancers are frequent, because the symptoms are similar to those of various benign diseases, such as urinary tract infection, kidney stones, and prostatic hyperplasia. For patients with high-grade malignancy of UCC, systemic chemotherapy is the standard treatment, providing a median survival of 15 months. Immunotherapy offers a new hope to patients with UCC that progresses after their initial systemic therapy [4]. The armamentarium for the treatment of advanced or metastatic UCC is expanding.

Growth arrest-specific 5 (GAS5), a long noncoding RNA (LncRNA A; ~0.7 kb), appears to not encode any functional proteins involved in modulating cell proliferation, apoptosis, drug resistance, invasion, metastasis, DNA repair, or epithelial-mesenchymal transition (EMT) [5,6,7]. GAS5 is mainly identified as a well-characterized tumor suppressor, and is downregulated in a wide range of human malignancies, including lung cancer, renal cell carcinoma, breast cancer, and bladder cancer, indicating that GAS5 could be utilized as a potential diagnostic hallmark [6,8]. Overexpression of GAS5 in hepatic stellate cells (HSCs) prevents liver fibrosis by targeting miR-222, resulting in cell cycle arrest and the inhibition of HSC activation [9]. Low expression of GAS5 is associated with a significantly higher risk for the early relapse and progression of non-muscle-invasive bladder cancer [8]. Studies of single nucleotide polymorphisms (SNPs) have investigated their role in the function of GAS5. SNPs in GAS5 rs145204276 Ins/Del and Del/Del genotypes were associated with a reduced risk of atherosclerosis, because they inhibit cell proliferation and stimulate the apoptosis of endothelial cells by targeting the GAS5/miR-21/PDCD4 signaling pathway [10]. Expression of GAS5 was upregulated in patients with acute myeloid leukemia (AML) and the GAS5 rs55829688 CC genotype; the promoter activity of GAS5 was increased through interaction with TP63, which in turn led to a worse prognosis for AML [11]. In a previous study, we identified that patients with prostate cancer and the Ins/Del or Del/Del genotype of GAS5 rs145204276 were at a significantly decreased risk of pathological lymph node metastasis compared with those with the Ins/Ins genotype (odds ratio (OR)  =  0.545; *p* = 0.043) [12]. Another report on bladder cancer indicated that patients in Iranian populations with the TG haplotype carriers of GAS5 (rs2067079 and rs6790) have a high risk of bladder cancer [13]. Whether GAS5 SNPs (rs145204276 and rs55829688) affect susceptibility to UCC remains unclear. In this case-control study, we identified the relationship between two GAS5 polymorphisms (rs145204276 and rs55829688) and clinical characteristics in Taiwanese patients with UCC.

## 2. Materials and Methods

### 2.1. Study Subjects and Ethics Statement

We enrolled 430 patients (271 men and 159 women) diagnosed with UCC and treated at Taichung Veterans General Hospital between 2010 and 2015. Healthy comparison groups (564 men and 296 women) with no history of or current cancer were also recruited. Before entering the study, all the participants completed a written questionnaire to disclose their demographic information. The study was certified by the Institutional Review Board (IRB) of Taichung Veterans General Hospital (IRB no. CF11094; 27 July 2011) prior to commencement, and informed consent was obtained from each participant. All the patients included in this study had undergone a radical cystectomy with pelvic lymph node dissection. Therefore, their T and N statuses were all determined by the radical cystectomy specimens. During the study period, neoadjuvant chemotherapies (NACs) were still not the routine practice before radical cystectomy in our institute. It was basically dependent on the physician’s preference to arrange for NAC or not. Therefore, in this study, we excluded all the participants with NAC to minimize the bias of survival analysis. All the patients with cN3 status received NAC, and consequently were excluded from the population. Whole blood samples were collected in vacutainer tubes containing ethylenediaminetetraacetic acid (EDTA) from the patients with UCC and the controls. The samples were inverted to mix and prevent clotting, immediately centrifuged, and stored at −80 °C until analysis [14,15].

### 2.2. Genomic DNA Isolation and the Determination of Genotypes

Frozen blood samples maintained on dry ice were transferred to an extraction laboratory, where they were thawed. The total genomic DNA was isolated from the samples using QIAamp DNA blood mini kits (Qiagen, Valencia, CA, USA) following manufacturer instructions for DNA isolation [16,17]. The purified DNA was suspended from the column in Tris-EDTA (TE) buffer (10 mM Tris pH 7.8 and 1 mM EDTA). We estimated DNA concentration by measuring absorbance at 260 nm with a spectrophotometer. The final preparation was stored at −20 °C and was subjected to quantitative polymerase chain reaction analysis. Sequencing analysis of GAS5 gene polymorphisms was performed using TaqMan SNP genotyping assays with the ABI StepOne Real-time PCR System, according to the supplier’s manual [12]. The polymorphisms were further analyzed using SDS version 3.0 (Applied Biosystems, Foster City, CA, USA).

### 2.3. GAS5 Expression Analysis of the Cancer Genome Atlas

Cohorts of the Cancer Genome Atlas (TCGA; URL: https://tcga-data.nci.nih.gov/tcga/), a landmark cancer genomics program, were used as our study validation cohorts for bladder urothelial carcinoma (BLCA), and the clinical significance of GAS5 in BLCA (*n* = 374) was assessed. Correlations between the mRNA levels of GAS5 in BLCA were also examined. GAS5 expression, classified into low expression (<median) and high expression (>median), were generated with respect to overall survival using the Kaplan-Meier method. Then *p*-values were calculated using a log-rank test [18].

### 2.4. Statistical Analyses

The genotypic distributions and clinical characteristics of each SNP in cases and healthy controls was compared through the chi-squared test. Fisher’s exact test and a Mann-Whitney U test were used to calculate the distributions of demographic characteristics and genotype frequencies. Logistic regression was used to calculate ORs and 95% confidence intervals (CIs) for the genotypic frequencies of GAS5 and various clinicopathological characteristics; *p* < 0.05 indicated statistically significant difference. All data were determined with SAS version 9.1, 2005 (SAS Institute Inc., Cary, NC, USA).

## 3. Results

The results of the statistical analyses of the demographic and clinicopathological characteristics of the participants are reported in Table 1. This study analyzed 430 cases (159 women and 271 men) with UCC and 860 healthy controls (296 women and 564 men) at Taichung Veterans General Hospital between 2010 and 2015. Significant distributional differences were observed in age (*p* < 0.001) between the control participants (mean age = 57.2 ± 10.0 years) and patients with UCC (mean age = 68.6 ± 11.8 years). No significant differences in distributions of gender or tobacco consumption between patients with UCC and the healthy controls were noted. At diagnosis, 195 patients (45.3%) had muscle-invasive urothelial carcinomas (pathologic stages pT2–pT4), 340 (79.1%) had a higher stage of tumor progression (T1–T4), and 51 (11.9%) and 14% (3.3%) of patients had lymph node metastasis (N1 + N2) and cancer spread to other parts (M1), respectively. The percentage of high-grade tumors was 87.7% (377 patients).

The distributions of genotype and allele frequencies of GAS5 polymorphisms among patients with UCC and the control participants are denoted in Table 2. In the recruited control group, the distribution of GAS5 genotypes revealed that the most frequent alleles were heterozygous Ins/Del for rs145204276 and homozygous T/T for rs55829688. We used a logistic regression test to analyze the genotypes and found no obvious differences in the allele and genotype frequencies of rs145204276 and rs55829688 SNPs in GAS5 between the patients with UCC and the healthy controls (Table 2).

To clarify the role of SNP GAS5 rs145204276 in the clinicopathologic status of patients with UCC, we calculated the distribution frequency of clinical statuses and frequency of GAS5 genotypes in 430 patients with UCC. No significant differences were noted in tumor pathologic stage, tumor size, lymph node metastasis, distant metastases, or histopathologic grading (Table 3). Next, we estimated genotype distributions of rs145204276 GAS5 gene polymorphisms among 159 female patients with UCC (Table 4). The patients with the deletion allele (Ins/Del or Del/Del genotype) for rs145204276 were at a significantly higher risk of larger tumor status relative to patients with the Ins/Ins genotype (OR  =  3.373; 95% CI 1.329–8.558; *p* = 0.009; OR  =  2.628; 95% CI 1.001–6.929; *p* = 0.048; Table 4).

**Figure 1:**
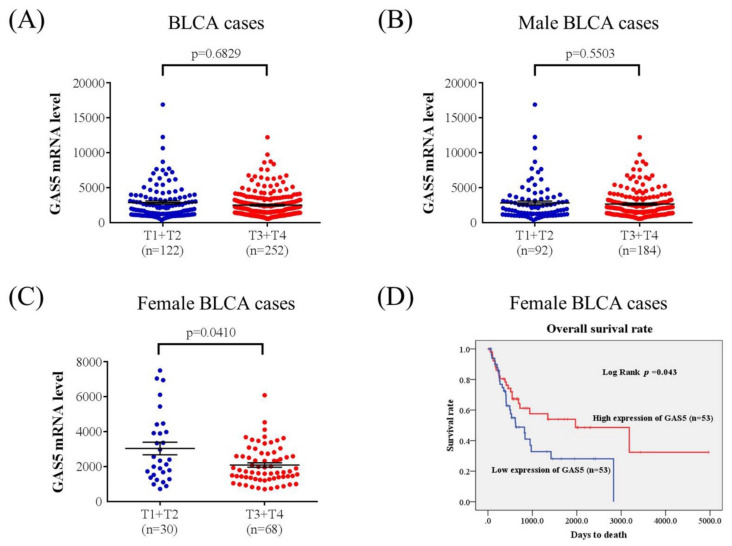
The clinical relevance of GAS5 levels in bladder urothelial carcinoma (BLCA) patients, obtained from the Cancer Genome Atlas (TCGA) database. (**A**) GAS5 mRNA levels in 374 bladder urothelial carcinoma tissues from TCGA were compared between patients with smaller tumors (T1–T2 status) and patients with larger tumors (T3–T4 status); (**B**) GAS5 mRNA gene expression levels in 274 male BLCA patients were compared according to tumor size status; (**C**) GAS5 mRNA gene expression levels in 98 female BLCA patients were compared according to tumor size status. Results were statistically significant by using Student’s *t*-test; (**D**) Survival analysis was performed for BLCA patients with high (red lines) and low (blue lines) GAS5 gene expression levels, using Kaplan-Meier analysis. The *p*-value was calculated using a log-rank test.

To further determine the clinical significance of GAS5 in UCC, 374 bladder UCC cases from the TCGA dataset were analyzed. No significant difference of GAS5 mRNA expression level was identified in BLCA between patients with smaller tumor sizes (T1–T2 status) and patients with larger tumor sizes (T3–T4 status; Figure 1A). In addition, levels of GAS5 in 274 male bladder urothelial carcinoma tissues did not correlate with tumor size status (Figure 1B). Significantly, lower GAS5 expression was observed in female patients with BLCA and larger tumor sizes than in patients with smaller tumor sizes (*p*  =  0.041; Figure 1C). Moreover, the results revealed that female patients with BLCA and low levels of GAS5 expression had significantly poorer overall survival than did those with high GAS5 expression (Figure 1D and Table 5).

## 4. Discussion

LncRNA GAS5 has attracted the attention of researchers in recent years, because it plays a key role in the development of tumors. GAS5 was reported to be aberrantly expressed in several cancers [8], and genetic susceptibility involving GAS5 plays a critical role in various cancer types [12,13]. Overexpression of GAS5 is strongly associated with the suppression of tumor growth and development, because it suppresses miRNA-106a-5p expression through the mammalian target of the rapamycin (mTOR) pathway in gastric cancer [19]. Upregulation of GAS5 has also been found to decrease cancer invasion [19], reverse EMT [5], and induce DNA damage [20]. Our present data provide evidence supporting the proposition that decreased mRNA expression levels of GAS5 in tumor tissues have a decreased overall specific survival in female BLCA patients (Figure 1).

Research identifying the specific GAS5 gene involved in hereditary UCC susceptibility may contribute to cancer progression and risk management. The genotypic polymorphisms of the GAS5 gene have been described. Carriers of the Ins/Del or Del/Del genotype of GAS5 rs145204276 are at low risk for pathological lymph node metastasis of prostate cancer in Taiwanese populations [12]. GAS5 polymorphisms rs2067079 and rs6790 serve as predictive biomarkers for platinum-based, concurrent, chemoradiotherapy-induced severe myelosuppression and neutropenia among patients with nasopharyngeal carcinoma [21]. LncRNAs GAS5 is generally identified as a tumor suppressor involved in various type of human cancers [6,19], but reports suggest that GAS5 has the ability to promote cancer [22]. The deletion allele of rs145204276 is significantly associated with an increased risk of hepatocellular carcinoma (HCC) and is positively correlated with higher levels of GAS5 in HCC tissue [23]. Individuals with the TG genotypes of GAS5 rs2067079 and rs6790 are at a significantly increased risk of bladder cancer compared with those with the wild-type genotype in Iranian populations [13]. This may suggest that the SNPs of GAS5 play different roles in the susceptibility to and progression of different types of cancer. Our study demonstrates that female patients with UCC and carrying the Ins/Del or Del/Del genotype of GAS5 rs145204276 have a higher risk of larger tumor status (Table 4). At diagnosis, among 91 female patients with UCC and carrying the Ins/Del or Del/Del genotype, the percentage of patients with larger high tumor status (T1–T4) reached 90.1% (Table 4). These results suggest that female patients with BLCA have decreased overall specific survival resulting from carrying the deletion allele for rs145204276.

The variant rs145204276, a 5 bp indel polymorphism shown as “-/AGGCA”, was located in the promoter region of GAS5. Many studies have demonstrated that carriers of GAS5 rs145204276 have an increased risk of various types of cancer, including gastric cancer [24] and HCC [23]. Furthermore, in one study, individuals with the genotype Del/Del of GAS5 rs145204276 had a smaller size of osteosarcoma tumor than those with Ins/Ins, and those with the genotype Del/Del rs145204276 had remarkably higher expression of GAS5 [25]. A recent study determined that the methylation frequency of the Del/Del genotype rs145204276 was significantly higher than that of either the Ins/Ins or Ins/Del genotype. This genotype may regulate GAS5 transcription activity by affecting the methylation of CpG islands in the promoter region, and thereby contribute to hepatocarcinogenesis [23].

## 5. Conclusions

In conclusion, polymorphic variants of GAS5 were associated with the clinicopathologic status of female patients with UCC. The data presented here demonstrate that carriers of the deletion allele of GAS5 rs145204276 are at a higher risk of larger tumor status than carriers of the wild type Ins/Ins genotype. Variations in GAS5 may be a reliable prognostic indicator of disease progression in female patients with UCC.

## Figures and Tables

**Table 1:** The distributions of demographical characteristics in 860 controls and 430 patients with urothelial cell carcinoma (UCC).

Variable	Controls (*n* = 860) *n* (%)	Patients (*n* = 430) *n* (%)	*p*-Value
Age (years)			
Mean ± S.D.	57.2 ± 10	68.6 ± 11.8	<0.001
Gender			0.365
Female	296 (34.4%)	159 (37.0%)	
Male	564 (65.6%)	271 (63.0%)	
Tobacco consumption			0.132
No	562 (65.3%)	299 (69.5%)	
Yes	298 (34.7%)	131 (30.4%)	
Stage			
Non muscle invasive tumor (pTa–pT1)		235 (54.7%)	
Muscle invasive tumor (pT2–pT4)		195 (45.3%)	
Tumor T status			
Ta		90 (20.9%)	
T1–T4		340 (79.1%)	
Lymph node status			
N0		379 (88.1%)	
N1 + N2		51 (11.9%)	
Metastasis			
M0		416 (96.7%)	
M1		14 (3.3%)	
Histopathologic grading			
Low grade		53 (12.3%)	
High grade		377 (87.7%)	

Student’s *t*-test or chi-squared test was used between controls and patients with UCC.

**Table 2:** Genotype distributions of GAS5 gene polymorphisms in 860 controls and 430 patients with UCC.

Variable	Controls (*n* = 860) *n* (%)	Patients (*n* = 430) *n* (%)	OR (95% CI)	AOR (95% CI)
rs145204276				
Ins/Ins	355 (41.3%)	191 (44.4%)	1.000	1.000
Ins/Del	388 (45.1%)	191 (44.4%)	0.915 (0.715–1.171)	0.948 (0.678–1.324)
Del/Del	117 (13.6%)	48 (11.2%)	0.763 (0.522–1.114)	0.737 (0.435–1.247)
Ins/Del + Del/Del	505 (58.7%)	239 (55.6%)	0.880 (0.696–1.111)	0.900 (0.654–1.238)
rs55829688				
TT	412 (47.9%)	208 (48.4%)	1.000 (reference)	1.000 (reference)
TC	354 (41.2%)	187 (43.5%)	1.046 (0.820–1.335)	0.948 (0.682–1.319)
CC	94 (10.9%)	35 (8.1%)	0.738 (0.483–1.125)	0.567 (0.304–1.055)
TC + CC	448 (52.1%)	222 (51.6%)	0.982 (0.779–1.237)	0.867 (0.633–1.188)

The odds ratios (ORs) with their 95% confidence intervals were estimated by logistic regression models. The adjusted odds ratio (AOR) with their 95% confidence intervals were estimated by multiple logistic regression models after controlling for age, gender, and tobacco consumption.

**Table 3:** Distribution frequency of the clinical status and growth arrest-specific 5 (GAS5) rs145204276 genotype frequencies in 430 UCC patients.

Variable	GAS5 (rs145204276)			
	Ins/Ins (%) (*n* = 191)	Ins/Del + Del/Del (%) (*n* = 239)	OR (95% CI)	*p* Value
Stage				
Non-muscle-invasive tumor	104 (54.5%)	131 (54.8%)	1.000 (reference)	
Muscle-invasive tumor	87 (45.5%)	108 (45.2%)	0.986 (0.673–1.444)	0.940
Tumor T status				
Ta	44 (23.0%)	46 (19.3%)	1.000 (reference)	
T1-T2	81 (42.4%)	116 (48.5%)	1.370 (0.830–2.262)	0.218
T3-T4	66 (34.6%)	77 (32.2%)	1.116 (0.658–1.892)	0.684
Lymph node status				
N0	166 (86.9%)	213 (89.1%)	1.000 (reference)	
N1 + N2	25 (13.1%)	26 (10.9%)	0.811 (0.451–1.455)	0.481
Metastasis				
M0	186 (97.4%)	230 (96.2%)	1.000 (reference)	
M1	5 (2.6%)	9 (3.8%)	1.456 (0.480–4.418)	0.505
Histopathologic grading				
Low grade	23 (12.0%)	30 (12.6%)	1.000 (reference)	
High grade	168 (88.0%)	209 (87.4%)	0.954 (0.534–1.703)	0.873

The odds ratio (OR) with their 95% confidence intervals were estimated by logistic regression models.

**Table 4:** Distribution frequency of the clinical status and GAS5 rs145204276 genotype frequencies in 159 female UCC patients.

Variable	GAS5 (rs145204276)			
	Ins/Ins (%) (*n* = 68)	Ins/Del + Del/Del (%) (*n* = 91)	OR (95% CI)	*p* Value
Stage				
Non muscle invasive tumor	38 (55.9%)	46 (50.5%)	1.000 (reference)	
Muscle invasive tumor	30 (44.1%)	45 (49.5%)	1.239 (0.659–2.329)	0.505
Tumor T status				
Ta	17 (25.0%)	9 (9.9%)	1.000 (reference)	
T1-T2	28 (41.2%)	50 (54.9%)	3.373 (1.329–8.558)	0.009 *
T3-T4	23 (33.8%)	32 (35.2%)	2.628 (1.001–6.929)	0.048 *
Lymph node status				
N0	60 (88.2%)	81 (89.0%)	1.000 (reference)	
N1 + N2	8 (11.8%)	10 (11.0%)	0.926 (0.345–2.486)	0.879
Metastasis				
M0	68 (100%)	88 (96.7%)	1.000 (reference)	
M1	0 (0.0%)	3 (3.3%)		0.131
Histopathologic grading				
Low grade	9 (13.2%)	5 (5.5%)	1.000 (reference)	
High grade	59 (86.8%)	86 (94.5%)	2.624 (0.837–8.223)	0.088

Bold font indicates statistical significance (* *p* < 0.05); The odds ratios (ORs) with their 95% confidence intervals were estimated by logistic regression models.

**Table 5:** Multivariate survival analysis of GAS5 expression and tumor T status in 106 female bladder urothelial carcinoma (BLCA) patients, according to the Cox proportional hazards regression model.

Variable	Hazard Ratio (95% Confidence Interval)	*p*-Value
Tumor T status (T1 + T2 vs. T3 + T4)	3.355 (1.604–7.015)	<0.001
GAS5 expression (low vs. high)	0.491 (0.271–0.888)	=0.019
